# Targeted ablation of the distal Purkinje-myocardium interface for premature ventricular complex–induced cardiomyopathy by delayed Purkinje conduction-induced re-excitation: a case report

**DOI:** 10.1093/ehjcr/ytaf335

**Published:** 2025-07-16

**Authors:** Rachel M A ter Bekke, Tom van Rooij, Paul G A Volders, Mélèze Hocini

**Affiliations:** Department of Cardiology, Cardiovascular Research Institute Maastricht (CARIM), Maastricht University Medical Center+, PO Box 5800, Maastricht, 6202 AZ, The Netherlands; Biosense Webster, Johnson & Johnson MedTech, Computerweg 14, 3821 AB, Amersfoort, The Netherlands; Department of Cardiology, Cardiovascular Research Institute Maastricht (CARIM), Maastricht University Medical Center+, PO Box 5800, Maastricht, 6202 AZ, The Netherlands; Cardio-Thoracic Unit, Bordeaux University Hospital (CHU), Pessac F-33600, France; IHU Liryc, Electrophysiology and Heart Modeling Institute, Fondation Bordeaux Université, Pessac-Bordeaux F-33600, France

**Keywords:** His-Purkinje system, Purkinje–muscle interactions, Ventricular arrhythmia, Cardiomyopathy, Catheter ablation, Case report

## Abstract

**Background:**

In structurally normal hearts, premature ventricular complexes (PVCs) are primarily driven by enhanced automaticity or afterdepolarization-dependent triggered activity. Traditionally, re-entrant excitation has only been associated with cardiac conditions involving scar formation, such as post-myocardial infarction or cardiac sarcoidosis.

**Case summary:**

We present a case of an asymptomatic 28-year-old patient with a high burden of monomorphic PVCs originating near the posteromedial papillary muscle. Left ventricular (LV) dilatation with reduced systolic function (ejection fraction 45%) was diagnosed as PVC-induced cardiomyopathy, given the absence of fibrosis and coronary artery disease. During an electrophysiological study, a 2-cm^2^ region was identified where abnormal Purkinje potentials (P1), exhibiting markedly reduced conduction velocity (0.88 mm/ms), consistently followed the rapid conduction via the left posterior fascicle (LPF). Local activation time velocity vectors of P1 pinpointed the earliest abnormal Purkinje activation at the proximal LPF. The impulse excited the distal one-third of the interventricular septum before conducting retrogradely to the LPF. Radiofrequency ablation targeted at the Purkinje-myocardial pivot point successfully eliminated the PVCs, restoring LV systolic function at follow-up.

**Discussion:**

Even in the absence of structural heart disease, delayed anterograde Purkinje conduction can facilitate monomorphic PVCs via re-excitation. This highlights the potential for targeted ablation at the distal Purkinje network as an effective treatment strategy.

Learning points

- Delayed Purkinje-fascicular conduction-induced re-excitation is a well-recognized mechanism underlying fascicular ventricular tachycardia; however, its role in Purkinje-related premature ventricular complex formation has not been previously reported.
- Even in the absence of apparent structural heart disease, delayed anterograde Purkinje conduction can facilitate monomorphic premature ventricular complexes via re-excitation.
- Recognizing delayed Purkinje conduction-induced re-excitation is important as targeted ablation at the distal Purkinje-myocardium interface may be indicated, avoiding risk of ablation-induced proximal conduction block.

## Introduction

Premature ventricular complexes (PVCs) can occur in the context of overt heart disease, but also in the absence of apparent structural or primary electrical abnormalities. The electrophysiological mechanisms that underlie arrhythmogenesis are abnormal automaticity, afterdepolarization-dependent triggered activity, and re-entry. Purkinje-related PVCs are mostly attributed to enhanced automaticity that may result in parasystole, often with variable coupling intervals (CIs).^1^ Conversely, PVCs originating from the outflow-tract regions are typically due to catecholamine-induced delayed afterdepolarizations and resulting triggered activity. In rare instances, re-entrant excitation underlies the occurrence of PVCs as has been described in conditions associated with structural heart disease like chronic myocardial infarction^2^ or cardiac sarcoidosis.^3^ Here, we present a unique case of delayed Purkinje conduction-induced re-excitation, leading to PVC-induced cardiomyopathy, which was successfully treated by targeted ablation at the distal Purkinje-myocardium interface.

## Summary figure

**Figure:**
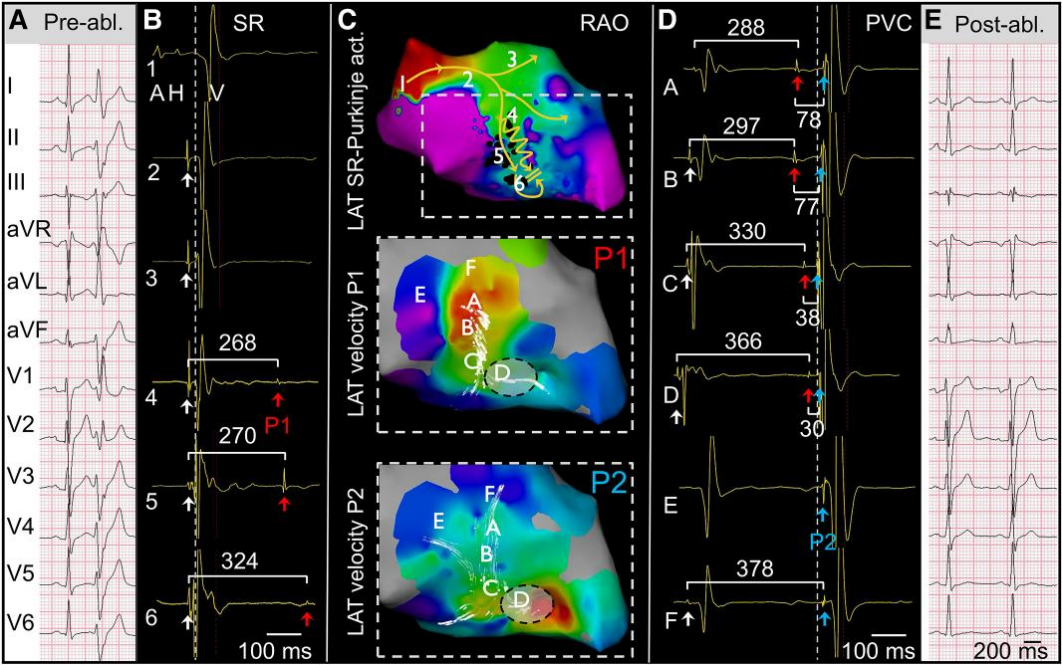


## Case description

**Figure 1:**
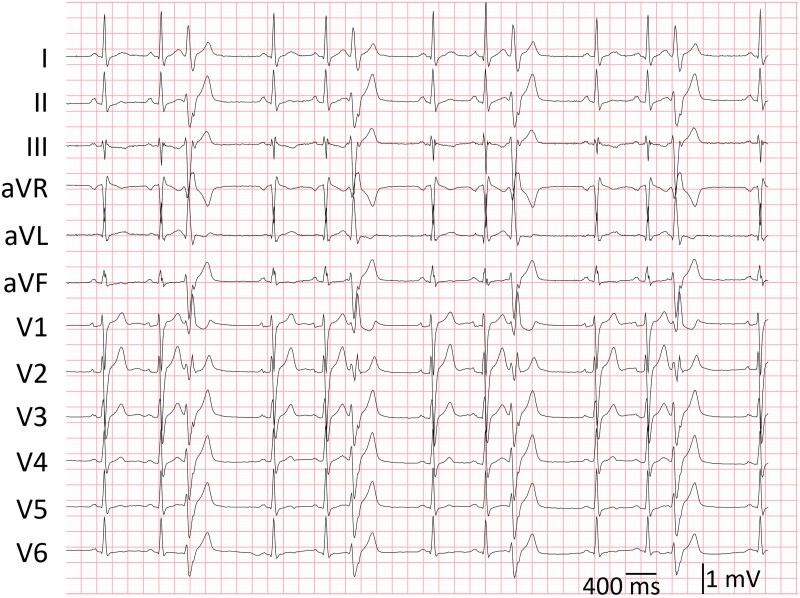
Twelve-lead electrocardiogram of sinus rhythm with monomorphic premature ventricular complexes in trigeminy prior to ablation. The premature ventricular complexes had a right bundle branch block morphology, a precordial transition at V3, and a left anterior hemiblock, suggesting an exit from the inferoseptal papillary muscle.

A 28-year-old previously healthy man was referred to our centre because of frequent monomorphic PVCs (in trigeminy, burden 21%–30%, measured on two Holter recordings; *Figure 1*). Besides minor fatigue, he reported no palpitations, shortness of breath, dizziness, or syncope. The PVCs had a right bundle branch block morphology with a superior axis and transition at lead V3, suggesting an exit from the inferoseptal papillary muscle (*Figure 1*). The CIs varied between 300 and 442 ms without signs of parasystole. No sustained ventricular tachycardia (VT) was noted. Pre-procedural cardiac magnetic imaging (MRI) revealed left ventricular (LV) dilatation with a mildly reduced systolic function (ejection fraction 45%). There was no delayed gadolinium enhancement nor presence of a myocardial false tendon. Cardiac computed tomography demonstrated no signs of significant coronary artery disease. Hence, a PVC-induced cardiomyopathy was diagnosed. Metoprolol 50 mg and flecainide 200 mg inadequately suppressed the PVCs; therefore, catheter ablation was planned.

**Figure 2:**
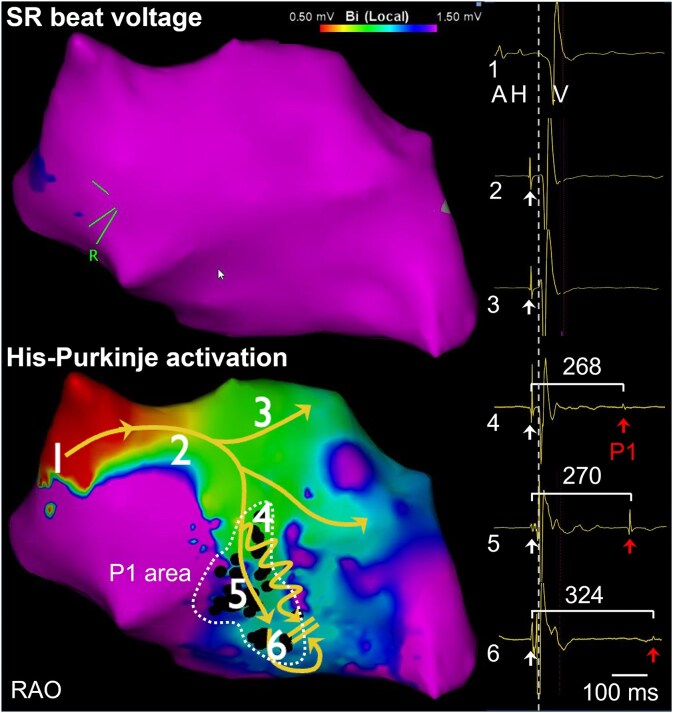
Electrophysiological characterization through invasive mapping of ventricular myocardial and Purkinje activation in sinus rhythm. Top left panel: Normal peak-to-peak bipolar voltage map displayed in right anterior oblique view. Lower left panel: Activation map of His-Purkinje activation during sinus rhythm showing normal anterograde specialized conduction system excitation (white arrows) starting from the His-bundle region (1), progressing along the proximal left bundle branch (2), left posterior fascicle (5–6), and anterior right fascicle (3). The dashed vertical line represents the earliest QRS onset recorded on the surface electrocardiogram. Aberrant, slowly conducting Purkinje re-excitation (P1, red arrows, proximal coupling interval 268 ms) was recorded at the proximal arborization of the left posterior fascicle. Electrograms at locations 1–6 are displayed on the left side.

**Figure 3:**
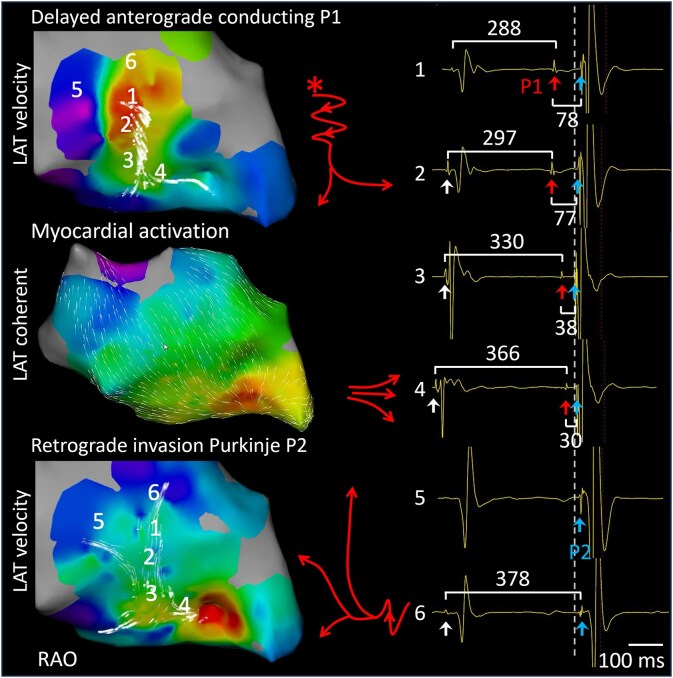
Electrophysiological characterization through invasive mapping of ventricular myocardial and Purkinje activation during premature ventricular activation. Top left panel: Local activation time (LAT) velocity vectors of P1 displaying the earliest proximal onset (marked by asterisk) with anterograde, delayed conduction properties (threshold 0.88 mm/ms). Middle panel: Local activation time with coherent vectors showing the earliest ventricular myocardial activation at the distal one-third of the interventricular septum. Lower panel: Local activation time velocity vectors of P2, showing retrograde invasion of the left posterior fascicle. A schematic representation of the electrical excitation is provided. Intracardiac electrograms at locations 1–6 are displayed on the left side, showing the relation between the P1 (red arrows) and P2 (blue arrows) activations, at position 1–6. The dashed vertical line represents the earliest QRS onset recorded on the surface electrocardiogram.

The unifocal PVCs were abundantly present during the electrophysiological study. As per our workflow, the THERMOCOOL SMARTTOUCH® D/F catheter (Biosense Webster Inc., Diamond Bar, USA) was advanced into the LV via retrograde aortic approach and electroanatomical mapping was performed using the CARTO® 3 System Version 8 with local activation time velocity vector mapping. In sinus rhythm, endocardial bipolar peak voltage mapping showed normal LV septal amplitudes (>1.5 mV, *Figure 2*, top) with fast anterograde conduction over the His-Purkinje system, including the left posterior (LPF) and anterior fascicle and an intermediate branch (*Figure 2*, bottom). Interestingly, a 2-cm^2^ region near the proximal arborization of the LPF was identified where delayed Purkinje potentials (*Figure 2*, P1, red arrows; black dots) consistently followed the preceding fast-conducting LPF-driven myocardial activation (for ripple map, see Supplementary material online, *Video S1*). The QRS-P1 CI varied between 265 and 282 ms. Shorter-coupled P1 Purkinje activity did not result in ventricular depolarization, whereas P1 activation at longer CI did propagate and re-excite the ventricular myocardium at the distal Purkinje-myocardium interface at the apical part of the interventricular septum (*Figure 3*). Local activation time velocity vectors of P1—compression 3, threshold 0.88 mm/ms—pinpointed the earliest abnormal Purkinje activation at the proximal LPF, which conducted anterogradely with a markedly reduced conduction velocity, measured by dividing the time difference between P1 proximal to P1 distal by the distance (CV, 0.88 mm/ms; *Figure 3*, top left panel).

After ventricular myocardial excitation (*Figure 3*, middle panel), the LPF was retrogradely invaded by a re-excitation wave (*Figure 3*, lower panel). P2 exhibited a normal CV of 1.85 mm/ms, depicted with local activation time velocity threshold 0.88 mm/ms and compression level 4. Additional pacing manoeuvres like overdrive pacing to confirm the re-entrant mechanism do not apply for a solitary re-entry loop and were not performed. No local fragmentation or split potentials were found. A multi-electrode grid-like or eight-spline catheter was not introduced because of potential catheter-induced PVCs in the vicinity of the distal conduction system.

**Figure 4:**
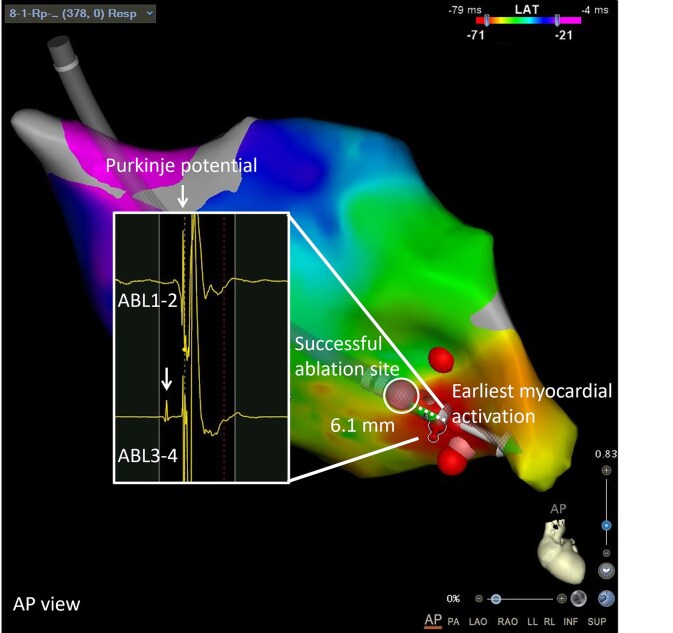
Targeted ablation at the Purkinje-myocardial pivot point in antero-posterior view. The site of successful ablation was 21 mm distal from the earliest P1 activation and 6.1 mm proximal from the earliest ventricular activation site.

**Figure 5:**
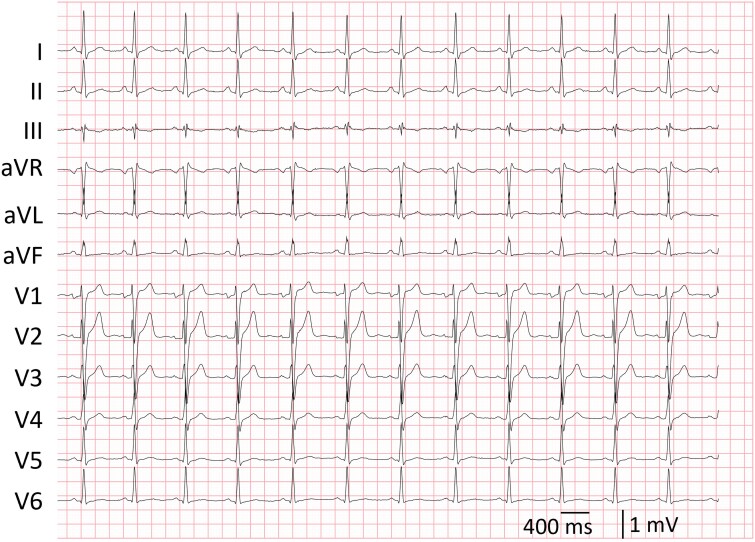
Post-ablation 12-lead electrocardiogram displaying normal sinus rhythm with minimal rightward axis deviation of the terminal QRS, but no premature ventricular complexes.

Radiofrequency energy delivery (30 W, flow rate 30 mL/min, mean contact force 7 g, and mean ablation index 505) was directed at the Purkinje-myocardial pivot point (*Figure 4*), not at the site of earliest Purkinje activation to avoid LPF damage, eliminating all PVCs. The site of successful ablation was 21 mm distal from the earliest P1 activation and 6.1 mm proximal from the earliest ventricular activation site. Anterograde P1 activation remained present, but with consistent distal Purkinje-myocardium block. Post-ablation, a slight rightward axis deviation of the terminal QRS persisted (*Figure 5*). All antiarrhythmic medication was discontinued. At 6-month follow-up, PVCs remained absent on 24-h Holter monitoring and electrocardiogram recording. Left ventricular ejection fraction normalized completely.

## Discussion

Whereas delayed Purkinje-fascicular conduction-induced re-excitation is a well-recognized mechanism underlying fascicular ventricular tachycardia,^4–7^ its role in Purkinje-related PVC formation has not been previously reported. In the absence of apparent structural abnormalities or abnormal anatomical pathways, re-entrant excitation requires two adjacent regions with significantly different conduction velocities and/or refractory periods to facilitate unidirectional block. In this 28-year-old patient, we consistently observed abnormal Purkinje P1 potentials following rapid anterograde activation of the LPF in the proximal LPF region. Aberrant Purkinje-fibre activation occurred at varying CIs with significantly reduced conduction velocities of 0.8 mm/ms (compared to normal velocities of 2–3 mm/ms).^8^

The timing of these abnormal activations and the short CI suggest that delayed conduction—potentially related to microstructural fibrosis—contributed to PVC formation, although an afterdepolarization-triggered mechanism cannot be fully ruled out. Shorter-coupled Purkinje excitations were blocked distally, likely due to longer refractoriness at the gate^9,10^ or retrograde invasion of the slowly conducting fascicle by preceding normal Purkinje activation (*Figure 3*, top). In contrast, longer-coupled Purkinje excitation propagated successfully, activating the distal ventricular myocardium and re-entering the normal LPF retrogradely (*Figure 3*, bottom).

Unlike the traditional ablation strategy for focal PVCs, where the earliest site of local activation is targeted, delayed Purkinje conduction-induced re-excitation was effectively ablated distally, near the Purkinje-myocardial pivot point. Our findings underscore the efficacy and safety of targeted ablation at the distal Purkinje-myocardium junction for functional abnormalities confined to the Purkinje system. A similar approach, ablating the latest local activation, was reported by Shinoda *et al.*^3^ in a case of re-entrant excitation in cardiac sarcoidosis with structural abnormalities. Whether microscopic fibrosis, undetectable with MRI or invasive mapping, has contributed to arrhythmogenesis remains elusive.

## Conclusion

Delayed anterograde conduction properties of the Purkinje system can promote the development of monomorphic PVCs through re-excitation, with retrograde concealed penetration into the specialized conduction system. Recognizing this mechanism allows for limited ablation at the distal Purkinje-myocardium junction, minimizing the risk of iatrogenic proximal conduction block.

## Supplementary Material

ytaf335_Supplementary_Data

## Data Availability

The data underlying this case report can be made available upon reasonable request to the corresponding author.

## Lead author biography

Dr Rachel M.A. ter Bekke is a clinical–experimental cardiologist–electrophysiologist with a fascination for ventricular arrhythmogenesis, including genetics and multimodality image integration to investigate arrhythmia formation and to tailor (non)invasive treatment in the structurally normal and diseased heart.
